# Deep learning for appendicitis: development of a three-dimensional localization model on CT

**DOI:** 10.1007/s11604-025-01834-1

**Published:** 2025-07-16

**Authors:** Taku Takaishi, Tatsuya Kawai, Yoshimasa Kokubo, Takumi Fujinaga, Yoshinao Ojio, Tatsuhito Yamamoto, Kana Hayashi, Yusei Owatari, Hirotaka Ito, Akio Hiwatashi

**Affiliations:** 1 Department of Radiology, Nagoya City University Graduate School of Medical Sciences, Kawasumi Mizuho-Cho, Mizuho-Ku, Nagoya, Aichi 467-8602 Japan; 2 Department of Radiology, Nagoya City University West Medical Center, Nagoya, Japan; 3 IT Solution Division, Medical Systems Business Div, FUJIFILM Corporation, Tokyo, Japan

**Keywords:** Deep learning, CT, Appendicitis

## Abstract

**Purpose:**

To develop and evaluate a deep learning model for detecting appendicitis on abdominal CT.

**Materials and methods:**

This retrospective single-center study included 567 CTs of appendicitis patients (330 males, age range 20–96) obtained between 2011 and 2020, randomly split into training (*n* = 517) and validation (*n* = 50) sets. The validation set was supplemented with 50 control CTs performed for acute abdomen. For a test dataset, 100 appendicitis CTs and 100 control CTs were consecutively collected from a separate period after 2021. Exclusion criteria included age < 20, perforation, unclear appendix, and appendix tumors. Appendicitis CTs were annotated with three-dimensional bounding boxes that encompassed inflamed appendices. CT protocols were unenhanced, 5-mm slice-thickness, 512 × 512 pixel matrix. The deep learning algorithm was based on faster region convolutional neural network (Faster R-CNN). Two board-certified radiologists visually graded model predictions on the test dataset using a 5-point Likert scale (0: no detection, 1: false, 2: poor, 3: fair, 4: good), with scores ≥ 3 considered true positives. Inter-rater agreement was assessed using weighted kappa statistics. The effects of intra-abdominal fat, periappendiceal fat-stranding, presence of appendicolith, and appendix diameter on the model’s recall were analyzed using binary logistic regression.

**Results:**

The model showed a precision of 0.66 (87/132), a recall of 0.87 (87/100), and a false-positive rate per patient of 0.23 (45/200). The inter-rater agreement for Likert scores of 2–4 was *κ* = 0.76. The logistic regression analysis showed that only intra-abdominal fat had a significant impact on the model’s precision (*p* = 0.02).

**Conclusion:**

We developed a model capable of detecting appendicitis on CT with a three-dimensional bounding box.

## Introduction

Appendicitis is a common cause of lower abdominal pain with a lifetime risk of 8.6% for males and 6.7% for females [1]. Given its high prevalence, diagnostic error or delay could result in severe complications, such as appendix perforation and sepsis, compromising patient care and safety [2]. CT is the preferred imaging modality to ultrasonography (US), especially in non-pregnant adult patients, who are at lower risk from ionizing radiation, due to its lower operator dependency, short scanning time over a wide area, and easier visualization of the appendix regardless of bowel gas or abdominal fat [3–6]. A meta-analysis by Terasawa et al. [7] demonstrated that CT outperforms US in the diagnosis of appendicitis in adult or adolescent patients, with a sensitivity of 94% [95% confidence interval (CI), 91–95] compared to 86% [95% CI, 83–88].

In recent years, artificial intelligence (AI) and machine learning have gained popularity in diagnostic radiology, with numerous algorithms have been developed for detecting various lesions [8]. However, detecting appendicitis remains a unique challenge for AI. The appendix’s tubular structure, variable anatomical positions, and resemblance to adjacent structures, such as cecum and ileum, complicate accurate localization [9]. While many studies have focused on classification models for diagnosing appendicitis [10], their limited ability to assist in localizing inflamed appendices leaves a gap in addressing clinically challenging cases. Only a handful of studies in this domain have attempted to incorporate detection models that provide precise volumetric localization [11].

In this study, we developed and evaluated a deep learning-based model for detecting appendicitis on CT scans. The model was based on the faster region convolutional neural network (Faster R-CNN) architecture and capable of predicting three-dimensional (3D) bounding boxes to locate inflamed appendices. To assess the feasibility of the model in diagnostic workflows, its performance was visually evaluated by experienced radiologists. Our research would contribute to the integration of AI tools into the clinical practice of abdominal radiology.

## Materials and methods

### Study design

**Fig. 1:**
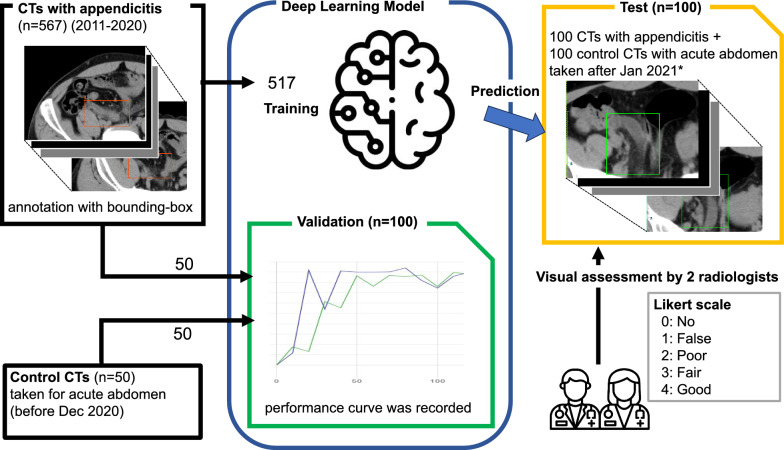
Overview of the study. The Likert scale of 3 or higher was regarded as a true-positive prediction

The overview is shown in Fig. 1. In this retrospective single-center study, a deep learning-based detection model was developed using CTs of appendicitis patients between January 2011 and December 2020. The model’s prediction was graded with a 5-point Likert scale by two board-certified radiologists using 100 appendicitis and 100 control CTs taken after January 2021.

Approval for this study was obtained from the internal Ethics Committee of our institution (reference number: 60-24-0146). Written informed consent was waived due to the retrospective design of the study based on preexisting images. This manuscript was written in accordance with the Checklist for Artificial Intelligence in Medical Imaging [12].

### Dataset

For the training and validation datasets, we retrospectively collected CTs of patients with acute appendicitis obtained at Nagoya City University Hospital between 1/1/2011 and 12/31/2020. Of the 567 eligible cases, 517 were randomly allocated to the training dataset. The remaining 50 cases were allocated to the validation dataset, combined with 50 control cases without appendicitis. These control cases were collected by reviewing consecutive CTs performed for acute abdomen before 12/31/2020, in reverse chronological order, until 50 eligible cases were obtained. For the test dataset, 100 CTs of appendicitis cases and 100 CTs of non-appendicitis cases with acute abdomen were consecutively collected after 1/1/2021. The diagnosis of appendicitis was confirmed on both radiology reports and electronic medical records. The exclusion criteria were patient age under 20 years, duplicate patients (the earliest scan was retained), perforated appendicitis, unclear appendicitis location, or appendix tumor pathologically identified after surgery.

Regarding the sample size for test dataset, we set a type I error rate of 5% (α = 0.05) and an acceptable absolute error of ± 5%. Assuming a recall of approximately 80% based on the previous literature [13], the sample size calculation indicated n = 62 [14, 15]. Thus, the number of appendicitis cases in the test dataset was set at 100 to exceed this threshold.

### Imaging protocols

All CTs included in this study were unenhanced, 5-mm-thick axial CTs. They were acquired using three different scanners: SOMATOM Definition Flash and SOMATOM Force (Siemens, Erlangen, Germany), and Aquilion ONE (Canon Medical Systems, Otawara, Japan). The imaging parameters were as follows: 120 kVp tube voltage, 100–800 mA tube current (adjusted via automatic exposure control), 0.6–1.2 helical pitch, 0.25–0.5 s rotation time, 512 × 512 pixel matrix. The CT dose index volume ranged from 6.8 to 29.1 mGy, and dose length product ranged from 264 to 1540 mGy·cm. The multi-slice images were cropped for the abdominopelvic section. CT reconstruction was performed using a vendor-specific soft-tissue kernel with a window level of 50 Hounsfield units (HU) and a window width of 400 HU.

To ensure patient privacy and comply with ethical standards, all CT data were fully de-identified in accordance with the Health Insurance Portability and Accountability Act privacy rule [16]. Patient identifiers, including names, dates of birth, and medical record numbers, were removed from the digital imaging and communications in medicine files.

### Annotation

For the appendicitis CTs in the training and validation datasets, a resident (Y.K. with 3 years of experience in diagnostic radiology) manually annotated the appendix with a 3D bounding box. While the appendix is a tubular structure with various shapes, the bounding box was designated to encompass the entire volume of the appendix from the appendiceal base to the distal portion. In addition, difficult cases with unclear appendicitis locations were reviewed in consultation with a board-certified radiologist (T.T. with 8 years of experience in diagnostic radiology). Normal appendices in the control CTs were not annotated.

### Model development

**Fig. 2:**
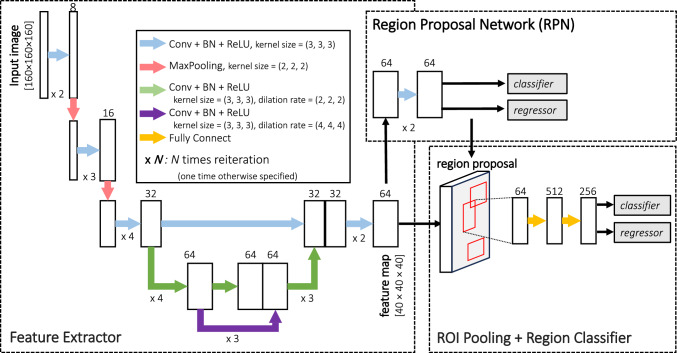
Pipeline of the deep learning model. Conv: convolution, BN: batch normalization, ROI: region of interest

Deep learning was conducted using the cloud-based AI development service “SYNAPSE Creative Space” (Fujifilm Corporation). As shown in Fig. 2, the pipeline was based on the Faster R-CNN architecture [17]. The details of the deep learning process are explained in the next paragraph.

As described in the Imaging Protocol section, the obtained CT image in this study consisted of anisotropic voxels, with a z-axis voxel length of 5 mm, an x, y-axis voxel length equal to the field-of-view divided by 512 (typically 0.6–0.8 mm per pixel), and the attenuation value ranged between -225 and 175 HU. These CT images were resampled to 2-mm isotropic voxel volumes using bilinear interpolation preserving the aspect ratio, and the attenuation value [ – 225, 175] was linearly rescaled to a range [0, 1]. Subsequently, the resampled volumes were randomly cropped to [160 × 160 × 160] (= 320-mm cube), the default input size required by the SYNAPSE Creative Space. During training, the feature extractor downsampled each input volume [160 × 160 × 160] to a [40 × 40 × 40] feature map, which was passed to the region proposal network (RPN) and ROI pooling layers (Fig. 2). The RPN proposed candidate regions, and ROI pooling was applied to extract fixed-size [7 × 7 × 7] feature volumes. These were then passed to the region classifier, which used a shared fully connected head for both object classification (classifier) and bounding box regression (regressor). During post-processing, bounding boxes with side lengths ≥ 4 mm, aspect ratios < 10, and confidence scores ≥ 0.3 were retained. For the loss function, cross-entropy loss was applied to the classifier, and smooth L1 loss to the regressor. The batch size was set to 4. Training was performed using the momentum optimizer with a learning rate of 0.0025. Computation was carried out on Nvidia Tesla T4 GPUs (16 GB) and AMD EPYC 7V12 (Rome) CPUs (56 GB) for up to 60 h. The best-performing model was selected based on the harmonic mean of precision and recall (F1 score).

### Model evaluation

Two board-certified radiologists (T.Y. and K.H. with 11 and 14 years of experience in diagnostic radiology, respectively) who were not involved in the model’s development process were recruited to evaluate the model’s performance. They reviewed all the CTs in the test dataset and visually assessed the model’s predictions using a 5-point Likert scale (0–4) as follows:

- 5-point Likert scale for the model’s prediction.

0. **No detection.**
1. **False detection**: the AI detects other structures.
2. **Poor detection**: the AI detects only a limited part of the lesion (approximately less than 50%) or identifies a normal appendix in control patients.
3. **Fair detection**: the AI prediction is satisfactory enough to locate the lesion but misses peripheral portions or includes too much surrounding tissue.
4. **Good detection**: the AI appropriately locates the lesion.

As the primary outcomes, we evaluated the model’s precision and recall using the following formulae:

- $$\text{Precision} = \frac{TP}{TP + FP} = \frac{\text{number of correctly detected lesions}}{\text{number of the model's predictions}}$$
- $$\text{Recall} = \frac{TP}{TP + FN} = \frac{\text{number of correctly detected lesions}}{\text{number of reference standard lesions}}$$

These metrics are preferred terms in the machine learning field, corresponding to precision = positive predictive value (PPV), and recall = sensitivity, respectively, in the field of diagnostic radiology. TP represents true positives, FP false positives, and FN false negatives. TPs were defined as model predictions where both raters assigned a score of 3 or higher. Otherwise, the model’s predictions were deemed FP. Also, FP rate per patient and the causes of FP predictions were assessed.

### Supplemental analysis

**Fig. 3:**
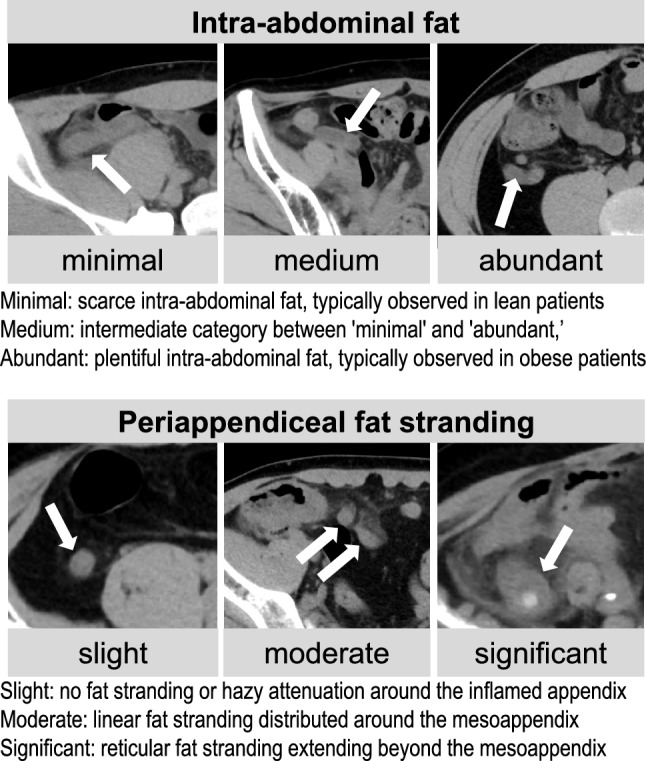
Examples of imaging feature rubrics and their definitions. White arrows indicate the location of appendicitis

The inter-rater variance of the Likert scale was assessed using the weighted kappa statistic [18]. For the appendicitis cases, the former radiologist (T.Y.) also evaluated the following imaging feature rubrics: intra-abdominal fat (minimal, medium, abundant), periappendiceal fat-stranding (slight, moderate, significant) (see Fig. 3), presence of an appendicolith (yes or no), and the appendix diameter (mm, measured as the maximum short axis). The intra-abdominal fat was evaluated because it may be related to the visibility of the appendix. The grading of fat-stranding was adapted and modified from the literature [19]. The effect of these rubrics on recall (= sensitivity) was assessed using binary logistic regression.

### Statistical analysis

For descriptive statistics, continuous variables were compared using the Mann–Whitney U test or the Kruskal–Wallis test (for comparing more than two groups), and categorical variables were compared using the Fisher’s exact test. The inter-rater variance was interpreted according to the criteria by Landis and Koch [20], where *κ*-values of 0.00–0.20 indicate slight agreement, 0.21–0.40 fair agreement, 0.41–0.60 moderate agreement, 0.61–0.80 substantial agreement, and 0.81–1.00 almost perfect agreement. The *p* values for predictors in the binary logistic regression analysis were calculated using the Wald tests. A two-sided *p* value of ≤ 0.05 was considered to indicate statistical significance. All statistical analyses were performed with the open-source statistics package R version 4.4.2 [21].

## Results

### Patient selection and demographics

**Fig. 4:**
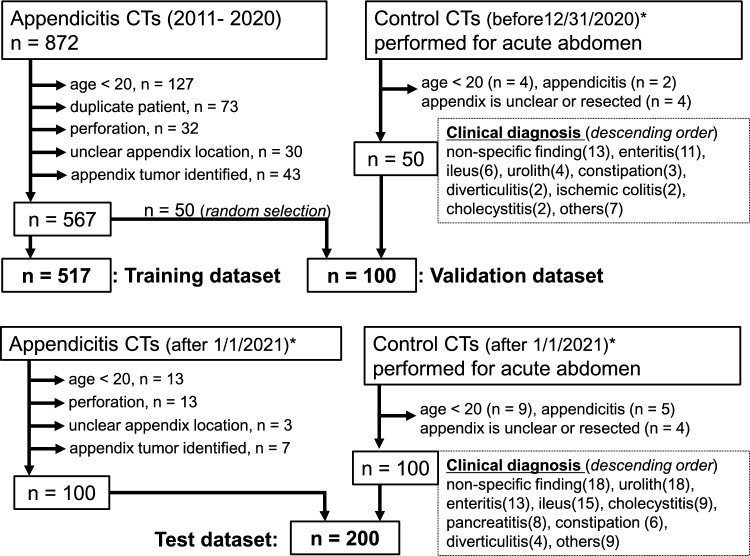
Flowchart of patient selection. *Eligible patients were consecutively collected backward or forward from a specific date until reaching the target number (50 or 100). Control CTs were not included in the training dataset because the deep learning architecture in this study utilized only positive cases as input features

Figure 4 shows the patient selection. Table 1 shows the demographics of patients. The ratio of [training]: [validation]: [test] was approximately 0.64: 0.12: 0.24. Ideally, a test should be done on outside sources [22], but access to data from other institutions was not feasible in this study. Thus, CT data from a completely separate time period were used as a test dataset.

**Table 1:** Patient demographic

	Training	Validation	Test	*p*
Appendicitis	*n* = 517	*n* = 50	*n* = 100	
Male: female	298: 219	32: 18	54: 46	0.69
Mean age (range)	50.3 (20–96)	45.1 (20–90)	50.7 (20–87)	0.13
Control	*n* = 0	*n* = 50	*n* = 100	
Male: female		20: 30	59: 41	0.037
Mean age (range)		59.0 (20–92)	58.2 (22–95)	0.64

### Model development

**Fig. 5:**
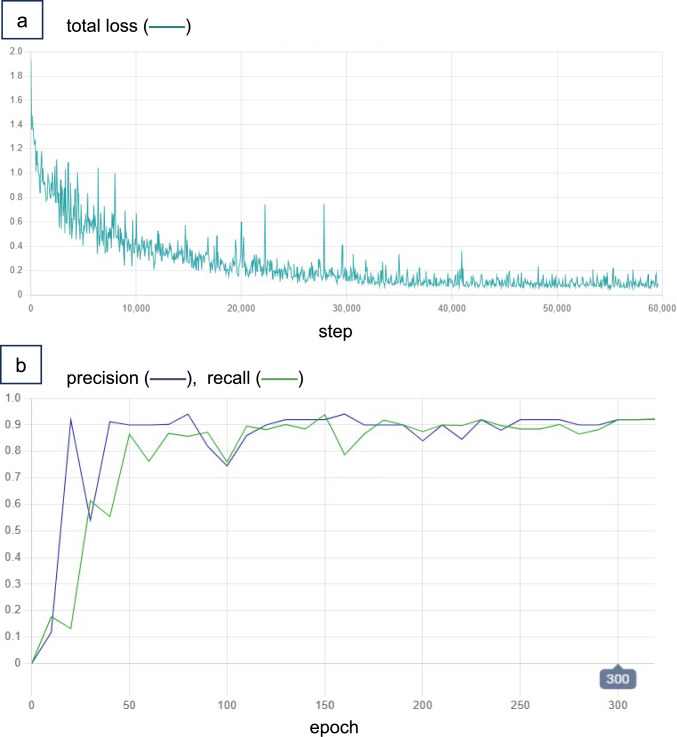
Model performance trends. **a** The graph illustrates the smooth convergence of the loss over the training steps. **b** The graph shows the progression of precision and recall on the validation dataset. The best performance was achieved at epoch 300, which was selected as the final model

Figure 5a shows the model’s learning curve. During training, the loss graph demonstrated smooth convergence. The total loss represents the sum of four components: smooth L1 and cross-entropy losses calculated at both the first-stage detection (region proposal network) and the second-stage classification (region classifier) processes, as outlined in Fig. 2. The model’s performance curve (Fig. 5b) plots precision and recall, which were recorded at 10-epoch intervals using the validation dataset. Internally, precision and recall were automatically calculated by defining true positives as cases where the intersection over union between the model-predicted bounding box and the resident-labeled bounding box was at least 0.125. This hyper-parameter was set arbitrarily for the purpose of validations. The model achieved its best metrics at epoch 300 (precision = 0.92, recall = 0.92, F1 score = 0.92), which was subsequently selected as the final model.

### Model evaluation

Two radiologists independently assessed the model’s predictions on the test dataset using the 5-point Likert scale. The primary outcomes of the model’s performance are summarized in Table 2. As described in the methods section, true positives were pre-defined as the model’s predictions where both raters assigned a score of 3 or higher. Otherwise, the model’s predictions were deemed FP.

**Table 2:** Primary outcome

Precision (= PPV)	Recall (= Sensitivity)	FP rate per patient
0.66 (87/132)	0.87(87/100)	0.23 (45/200)

*PPV* positive predictive value, *FP* false-positive

**Fig. 6:**
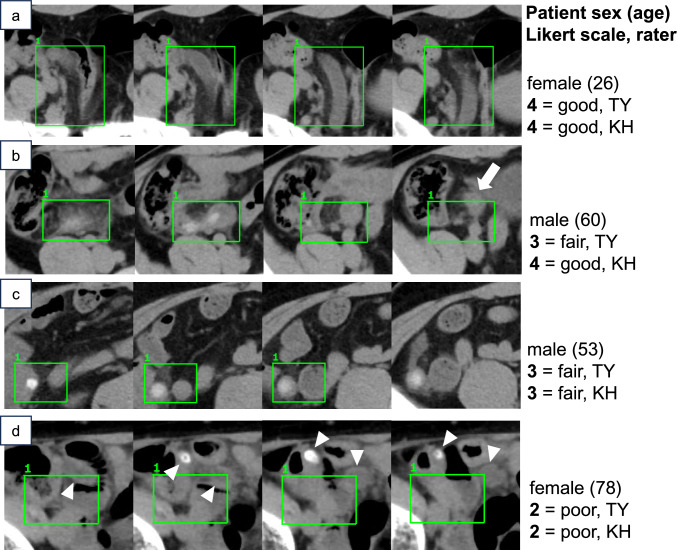
Examples of the model’s predictions and their Likert scales. Bounding boxes represent the model’s predictions. **a** The model appropriately encompasses the inflamed appendix. **b** The model omits the marginal portion of the appendix. **c** The model omits the distal portion (*white arrow*) but captures more than 50% of the appendix. **d** The model’s prediction slightly overlaps with the appendix (*arrowheads*). When there was a discrepancy between the two raters, the lower Likert score was adopted. The slice levels (distance in mm from the diaphragm) were as follows: a(280,285, 290,295), b(295,305,315,325), c(220,235,250,265), d(280,285,290,295)

The model achieved a precision of 0.66 (87/132), indicating that 66% of the model predictions were fair (score = 3) or good (score = 4). Recall was 0.87 (87/100), indicating the model’s ability to identify 87% of the appendicitis locations. Figure 6 shows examples of the model’s predictions. Among all the model predictions on the test dataset, FP rate per patient was 0.23 (45/200). FP prediction locations were as follows: ileum (*n* = 23), normal appendix (*n* = 8), cecum (*n* = 5), ascending colon (*n* = 4), descending colon (*n* = 2), poor detection* (*n* = 2), and right external iliac artery (*n* = 1). *Poor detection refers to cases where the model’s prediction partially overlapped with the appendix but was assigned a Likert score of 2 or lower by at least one radiologist.

### Supplemental analysis

The inter-rater agreement of the Likert scale (0–4) between the two radiologists was *κ* = 0.98 (almost perfect agreement). As the scoring of 2–4 was particularly ambiguous, we also performed the weighted kappa statistic by limiting the analysis to the Likert scores of 2–4, resulting in *κ* = 0.76 (substantial agreement).

**Fig. 7:**
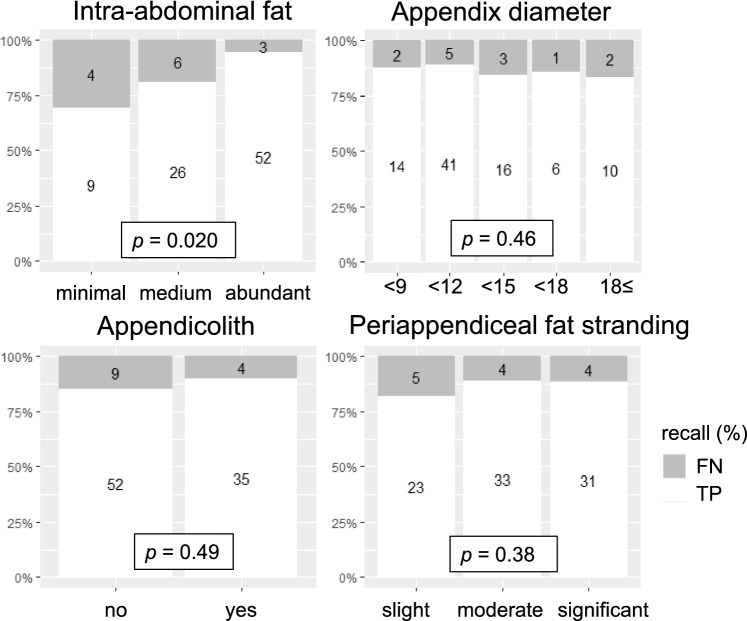
Model performance stratified by imaging feature rubrics

For appendicitis cases, the former radiologist (T.Y.) also evaluated the following imaging feature rubrics: intra-abdominal fat, periappendiceal fat-stranding, presence of an appendicolith, and the appendix diameter (mm). Figure 7 shows the result of binary logistic regression analysis. Among the rubrics, intra-abdominal fat was significantly associated with recall (*p* = 0.020), indicating that higher levels of fat facilitated the model’s detection of appendicitis. Other rubrics did not show statistically significant associations with recall.

## Discussion

In this study, we developed a deep learning model capable of predicting 3D bounding boxes to detect appendicitis. The model was based on Faster-RCNN architecture and was developed using 567 appendicitis CTs and 50 control CTs. The model’s performance was visually evaluated by two independent radiologists using 100 appendicitis CTs and 100 control CTs. Defining the TP prediction as cases where both the radiologists graded the Likert scale 3 (= fair) or 4 (= good), the model achieved a precision of 0.66, a recall of 0.87, and a FP rate of 0.23 per patient.

As shown in Table 3, several studies have proposed deep learning models for the diagnosis of appendicitis on CT. Most of these utilized classification frameworks to distinguish appendicitis from other conditions [23–25] or to assess severity [11, 26]. A study [26] also incorporated additional clinical features (e.g., CRP, neutrophil ratio) other than CT images. In contrast, only one prior study [11] developed a detection framework. This gap may stem from the difficulty of detection, given various appendix locations within the body and the myriad image manifestations of the inflamed appendix [27].

**Table 3:** Summary of prior studies

Study, year	Purpose	Model framework	Patient	Input features	Performance
Baştuğ et al. [11] 2024 (Turkey)	Segmentation	U-Net	299 patients (159 with AA)	Unenhanced 2.5 mm-slice axial abdominal CTs	Precision:87.0%, Recall: 87.0%, DSC:86.5%
Liang et al. [13] 2023 (China)	Classification of complicated or uncomplicated AA	Catboost Classifier using UCTransNet and PyRadiomics toolkit	Complicated AA 418 (training:236, test:182) uncomplicated AA 747 (training:464, test:283)	Unenhanced 3 mm-slice coronal CTs from 3 centers	Sensitivity: 70.2%, Specificity: 73.9%, PPV: 0.632, AUC: 0.799
Park et al. [23] 2023 (South Korea)	Classification of AA, diverticulitis, and normal	EfficientNet	AA 246 diverticulitis 254 with normal 338	Enhanced axial 2-5 mm-slice CTs	Sensitivity: 87.8%, PPV: 87.1%, Accuracy: 87.9%, AUC: 0.951
Noguchi et al. [24] 2021 (Japan)	Differentiating appendicitis from other acute abdomens	AlexNet	AA 485 non-AA 485	Enhanced 1 mm-slice axial CTs	Precision: NR, Recall:NR, Accuracy: 86.8%,
Rajpurkar et al. [25] 2020 (USA)	Classification of AA or non-AA	3D-ResNet	646 CTs (359 with AA)	Enhanced 1.25 mm axial CTs	AUC: 0.810, Sensitivity: 78.4%, Specificity: 66.7%,
Lin et al. [26] 2023 (Taiwan)	Classification of complicated or uncomplicated AA	Multilayer perceptron artificial neural network	Complicated AA:49, uncomplicated AA:362	CRP level, neutrophil ratio, CT findings (fat-stranding, appendicolith, and ascites)	AUC: 0.950, Sensitivity: 85.7%, Specificity: 91.7%

*AA* acute appendicitis, *NR* not reported, *DSC* dice similarity coefficient (= overlap-based metrics)

It is controversial whether a detection or a classification framework is more effective for improving clinical workflows. For diagnosing appendicitis, detection models may offer an advantage because the primary challenge in diagnosing appendicitis lies in identifying the inflamed appendix itself. Furthermore, identifying the appendix location and examining the associated inflammatory signs are crucial for risk evaluation and treatment strategy [28]. In contrast, classification models only provide an overall likelihood of appendicitis for the entire image, which may leave clinicians uncertain about whether the image truly represents appendicitis. For example, according to a multicenter study that assessed the effect of a chest radiograph AI model on 220 physicians’ diagnostic performance, local (bounding-box-indicated) AI explanations were associated with reduced reading time and better diagnostic accuracy compared to global (phenotype-based) AI explanations [29].

The annotation method in this study was bounding box, whereas the aforementioned detection model [11] employed a segmentation-based method. Segmentation provides higher annotation granularity by offering pixel-level localization of the inflamed appendix. On the contrary, the bounding box used in our study encompassed the inflamed appendicitis with a rectangular box, which may include surrounding tissues and therefore introduce localization obscurity. However, as suggested by our prior work [30], the detection performance of a bounding box model is comparable to, or even exceeds, that of a segmentation-based model. Furthermore, the detection approach is considerably less labor-intensive as it avoids the need for meticulous slice-by-slice contouring required in segmentation.

In this study, we used Faster R-CNN architecture, which has been applied in previous studies for detecting breast cancer, rib fractures, and knee osteoarthritis [31–33]. This architecture features a two-stage design in which a region proposal network (RPN) generates initial bounding boxes, and ROI pooling and a region classifier refines the boxes and improves object detection. In addition, we extended the bounding box to 3D. In this way, the model further enhanced localization accuracy by capturing spatial information across all axes.

As shown in Table 3, the use of contrast media varied across studies. Lane et al. [34] recommended non-enhanced helical CT as a highly accurate technique for diagnosing or excluding acute appendicitis, reporting a sensitivity of 96% and a specificity of 99%. On the other hand, Birnbaum et al. [35] advocated the use of contrast media for identifying the inflamed appendix, particularly in patients with mild appendicitis, limited mesenteric fat, or perforated appendicitis. In our study, we took the former position, using non-enhanced CT. At our institution, screening CTs for acute abdomen are typically performed without contrast media to avoid time constraints and side effects associated with intravenous contrast media injection. In fact, only a limited number of patients with appendicitis in this study underwent subsequent enhanced CT.

In the supplemental analysis, inter-rater agreement of the Likert scale was substantial or almost perfect based on the criteria by Landis and Koch [20]. Nevertheless, instead of using consensus reads, we adopted the lower score assigned by the two raters to ensure a conservative evaluation. The binary logistic regression analysis revealed that intra-abdominal fat was the only factor that significantly affected the model’s precision. This finding implies that intra-abdominal fat could affect the visibility of inflamed appendices on CT and influence the model’s performance. In contrast, other factors including periappendiceal fat-stranding, the presence of an appendicolith, and the appendix diameter did not show a significant effect on precision, suggesting that the model is robust against a broad range of appendicitis manifestations.

This study has several limitations. First, the evaluation of the model relied on a visual approach, which may contain subjective bias. Although a quantitative assessment of how well the predicted bounding boxes covered the true 3D regions of inflamed appendices would have been preferable, such an evaluation was considered impractical because it necessitates voxel-wise annotation of the tubular and convoluted morphology of the appendix. Nevertheless, since the deep learning model is expected to be used as an aid for radiologists in clinical practice, expert visual assessment may be acceptable. Second, this study was conducted at a single center with a retrospective design, which may limit the generalizability of our findings. Future research involving CT datasets from other institutions would help to better evaluate the model’s performance across diverse imaging conditions and patient demographics.

In conclusion, we developed a deep learning-based model capable of detecting appendicitis on CT with a 3D bounding box. This novel approach addresses the key diagnostic challenge of localizing inflamed appendices, which vary widely in location and imaging appearance. Compared to traditional classification models, this detection-focused approach provides precise localization, enhancing diagnostic confidence and facilitating risk evaluation and treatment planning. Additionally, the model exhibited robustness against diverse appendicitis manifestations, including varying periappendiceal fat-stranding and the presence of appendicoliths. By integrating this model into clinical workflows, there is potential to streamline the diagnostic process and improve outcomes for patients with acute abdomen.
